# The effects of the introduction of the single-family room in neonatal and paediatric intensive care on the outcomes of paediatric patients, families, staff, and organizations: a mixed method systematic review

**DOI:** 10.1186/s12913-025-13595-8

**Published:** 2025-10-24

**Authors:** Giulia Ottonello, Silvia Rossi, Nicoletta Dasso, Roberta Da Rin Della Mora, Simona Calza, Giuseppe Minniti Caracciolo, Ilaria Artuso, Simona Serveli, Fulvia Esibiti, Chiara Rebuffi, Stefano Parodi, Silvia Scelsi

**Affiliations:** 1 Health Professionals Direction, IRCCS Istituto G. Gaslini, Genova, Italy; 2 Scientific Direction, IRCCS Istituto G. Gaslini, Genova, Italy

**Keywords:** Nursing care, Single-family room, NICU, PICU, Outcome, Review, Family-centred care

## Abstract

**Background:**

The introduction of single-family rooms (SFRs) in neonatal (NICUs) and paediatric intensive care units (PICUs) enhances family involvement and transforms healthcare professionals’ approach to patient care. This shift promotes family-centred care while requiring adjustments in staff workflows and communication. Given the significant implications of this structural change, this review examines the effects of SFRs on paediatric intensive care patient outcomes, families, healthcare staff, and healthcare organizations.

**Methods:**

A systematic search was conducted in PubMed, CINAHL, PsycINFO, EMBASE, SCOPUS and Web of Science databases and grey literature. Studies published in English, Italian, and Spanish from 2009 to 2025 were included. The review adhered to the Johanna Briggs Institute methodology and PRISMA reporting guidelines to ensure rigor and transparency.

**Results:**

A total of 649 records were identified, with 30 studies meeting inclusion criteria. Findings suggest that SFRs in NICUs positively impact neonatal neurodevelopment, parental participation, and staff experiences. Infants in SFRs demonstrated improved neurobehavioral outcomes, better sleep regulation, higher exclusive breastfeeding rates, and shorter hospital stays. Increased parental presence strengthened parent-infant bonding, facilitated earlier milk expression, and reduced maternal stress. However, concerns were raised regarding potential sensory deprivation, which could affect long-term language and motor development. From the healthcare staff perspective, SFRs were associated with increased job satisfaction, improved family-centred care, and reduced noise-related stress. However, some nurses reported greater emotional strain, professional isolation, and communication challenges. Organizationally, SFRs improved workflow efficiency, enhanced infection control adherence, and reduced costs due to shorter hospital stays. Despite these benefits, only one study evaluating in PICUs setting was included, highlighting a significant gap in research.

**Conclusions:**

SFR NICUs offer substantial benefits in family-centred care, neurodevelopment, and hospital efficiency. Successful implementation requires careful planning to support staff well-being, promote collaboration, and address potential sensory deprivation. Healthcare administrators should ensure adequate training and emotional support of staff, and environmental enrichment strategies. Future research should focus on optimizing NICU designs to balance developmental advantages, parental engagement, staff satisfaction, and operational sustainability while expanding studies on PICU settings.

**Registration:**

This review is registered in PROSPERO (CRD42024501520).

## Background

Over the past two decades, the physical environment of neonatal (NICU) and paediatric intensive care units (PICU) has gained increasing attention for its impact on patient care, family well-being, and healthcare staff experiences. Traditional Open Bay (OB) models, where multiple patients share a single space, have long been the standard in NICUs and PICUs. However, a growing emphasis on family-centred care (FCC**)** has led to the introduction of single-family rooms (SFRs)**—**private hospital spaces that allow parents to remain with their child in a single room 24 h a day throughout hospitalization. This model is designed to create a home-like environment that fosters continuous parental involvement, strengthens parent-child bonding, and reduces stress for both patients and families [1].

Since 2009, SFRs have been progressively introduced in NICUs, with evidence suggesting significant benefits for neonatal health outcomes, parental well-being, and healthcare delivery [2, 3]. Inside these rooms, families have access to essential amenities such as sleeping areas, kitchens, and designated spaces for relaxation and play. Unlike the OB model, which exposes infants to excessive noise, light, and frequent disruptions, SFRs offer greater control over the environment, helping to regulate sensory stimulation and improve the overall care experience [4].

The existing literature indicates that SFRs have a positive impact on multiple aspects of care [2, 5]. Many studies have found that infants in SFRs experience better neurodevelopmental outcomes, improved sleep patterns, increased exclusive breastfeeding rates, and a shorter length of hospital stay. Parents who remain continuously present report stronger emotional bonding, earlier milk expression, and reduced stress and anxiety [6]. From a healthcare system perspective, SFRs have been associated with enhanced infection control, improved workflow efficiency, and potential cost savings due to a reduction in hospital days [5, 7].

Additionally, while healthcare staff generally acknowledge the advantages of SFRs, there are challenges associated with their implementation. Some studies report increased job satisfaction, improved family-centred care delivery [8, 9] and reduced noise and alarm fatigue among healthcare professionals [10]. However, others highlight concerns such as emotional strain, professional isolation, and reduced peer support, particularly among nurses [4].

A key challenge is maintaining effective communication and teamwork in a setting where staff interactions may be reduced due to the physical separation of patient rooms [8, 11]. Furthermore, there are concerns that sensory deprivation in SFRs—due to reduced exposure to hospital environment sounds and human interactions—might negatively influence language acquisition and motor development in infants, warranting further investigation [12–14]. However, while the benefits are well-documented in NICUs, research on SFRs in PICUs remains scarce. Given the different needs of critically ill paediatric patients—who often require more intensive monitoring and interventions—the feasibility and impact of SFRs in PICU settings are less understood [15]. Assessing how SFRs influence clinical outcomes, parental involvement, staff experiences, and healthcare organization dynamics is crucial to optimize intensive care environments.

The purpose of this review is to identify the impact of SFRs on paediatric patient outcomes, family engagement, staff experiences and organization, contributing to the ongoing development of evidence-based neonatal and paediatric intensive care models. Therefore, the research question was as follows: “What are the effects of the introduction of SFR in paediatric intensive care units and neonatal intensive care units on paediatric patients (0–18 years) outcomes, on family outcomes, on staff outcomes, and on organizational outcomes?”

## Methods

### Aim

The aim of this review is to identify the effects of the introduction of the SFR in NICU and PICU settings on paediatric patients (premature infants, newborns, infants, children and adolescents) outcomes, on the outcomes of families (intended as parents /caregiver in reference to the figures connected to the child who are physically in the room together with the patient) [16], on the outcomes of the clinical, care and transitional care staff, and on the organizational outcomes.

### Design

This is a mixed-methods systematic review with protocol registered in PROSPERO (International Prospective Register of Systematic Reviews)- CRD42024501520. Authors followed the mixed-methods systematic reviews guidelines (Mixed Methods Systematic Review of the Johanna Briggs Institute (JBI)). More protocols details can be found here [17].

### Inclusion and/or exclusion criteria

In this review, studies were included if they: (1) examined the impact of the single-family room (SFR) setup in neonatal and paediatric (0–18 years) intensive care units (NICUs and PICUs) on patients, families, healthcare staff, or organizations; (2) were primary studies using quantitative, qualitative, or mixed-methods designs; (3) were published in English, Italian, or Spanish; (4) were published from 2009 to June 2025 following the introduction of the SFR design in neonatal and paediatric intensive care units [2, 3].

Non-primary studies, including literature reviews and meta-analyses, were excluded.

### Search strategy

The search strategy was developed in collaboration with a librarian (CR), and details and keywords are outlined in Table 1. The terms, as well as indexed terms from each database, were incorporated into the search strategy, which was adapted for each database to ensure a comprehensive retrieval of relevant studies.

**Table 1:** Search strategy

DATABASE	Search Strategy
*PubMed*	((“single family room*“[Title/Abstract] OR “single family room*“[All Fields] OR “single room*“[Title/Abstract] OR “private room*“[Title/Abstract]) AND (“intensive care“[Title/Abstract] OR “intensive care unit*“[Text Word] OR “ICU“[Title/Abstract] OR “pediatric intensive care unit“[All Fields] OR “PICU“[Title/Abstract] OR “neonatal intensive care unit“[All Fields] OR “NICU“[Title/Abstract]) AND ((english[Filter] OR italian[Filter] OR spanish[Filter]) AND (allchild[Filter] OR newborn[Filter] OR allinfant[Filter] OR infant[Filter] OR preschoolchild[Filter] OR child[Filter] OR adolescent[Filter]) AND (2009:2025[pdat]))
*CINAHL*	AB (single family room OR single-family room OR single room OR private room) AND AB (intensive care OR intensive care unit OR ICU OR pediatric intensive care unit OR PICU OR neonatal intensive care unit OR NICU) Limiters - Publication Date: 20090101–20250630 Expanders - Apply related words; Apply equivalent subjects Narrow by Language: - english. italian, spanish Narrow by SubjectAge: - child, preschool: 2–5 years Narrow by SubjectAge: - child: 6–12 years Narrow by SubjectAge: - adolescent: 13–18 years Narrow by SubjectAge: - infant: 1–23 months Narrow by SubjectAge: - infant, newborn: birth-1 month Narrow by SubjectAge: - all infant Narrow by SubjectAge: - all child Search modes - Boolean/Phrase
*PsychInfo*	(single family room* or single-family room* or single room* or private room*) AND (intensive care or intensive care unit or ICU or pediatric intensive care unit or PICU or neonatal intensive care unit or NICU).mp. [mp = title, abstract, heading word, table of contents, key concepts, original title, tests & measures, mesh word] limit 3 to (human and english, italian, spanish language and (100 childhood < birth to age 12 yrs > or 120 neonatal < birth to age 1 mo > or 140 infancy < 2 to 23 mo > or 160 preschool age < age 2 to 5 yrs > or 180 school age < age 6 to 12 yrs > or 200 adolescence < age 13 to 17 yrs>) and yr="2009–2025”)
*Embase*	(‘single family room*’:ab, ti OR ‘single-family room’:ab, ti OR ‘single room’:ab, ti OR ‘private room’:ab, ti) AND (‘intensive care’:ab, ti OR ‘intensive care unit*’:ab, ti OR ‘pediatric intensive care unit*’:ab, ti OR ‘neonatal intensive care unit*’:ab, ti OR picu: ab, ti OR nicu: ab, ti) AND ([newborn]/lim OR [infant]/lim OR [child]/lim OR [preschool]/lim OR [school]/lim OR [adolescent]/lim) AND [2009–2025]/py AND ([english]/lim OR [italian]/lim OR [spanish]/lim)
*Scopus*	(TITLE-ABS-KEY (“single family room*” OR “single- family room” OR “single room” OR “private room”) AND TITLE-ABS-KEY (“intensive care” OR “intensive care unit” OR “ICU” OR “pediatric intensive care unit” OR “PICU” OR “neonatal intensive care unit” OR “NICU”)) AND PUBYEAR > 2008 AND PUBYEAR < 2024 AND (LIMIT-TO (EXACTKEYWORD, “Infant, Premature”) OR LIMIT-TO (EXACTKEYWORD, “Child”) OR LIMIT-TO (EXACTKEYWORD, “Infant Care”) OR LIMIT-TO (EXACTKEYWORD, “Nursing Staff, Hospital”) OR LIMIT-TO (EXACTKEYWORD, “Health Care Personnel”)) AND (LIMIT-TO (LANGUAGE, “English”, “Italian”, “Spanish”))
*Web of Science (WoS)*	(TS= (“single family room*” OR “single- family room*” OR “single room” OR “private room”)) AND TS=(“intensive care” OR “intensive care unit” OR ICU OR PICU OR NICU) Refined by: languages English, Italian, Spanish Publication date: 2009–2025

A comprehensive systematic search was carried out in December 2023 and then updated in June 2025 across six electronic databases: PubMed, CINAHL, PsycINFO, EMBASE SCOPUS and Web of Science. Grey Literature was explored. In addition, reference lists from relevant systematic reviews and other types of reviews were screened to identify any additional studies that may not have been retrieved in the initial search.

### Study selection and screening

All citations retrieved from the search were imported into EndNote Web, where duplicates were removed. The titles and abstracts of the studies were screened by five groups of researchers, each consisting of two members to determine eligibility for inclusion. Screening for full-text articles inclusion was assessed, and reasons for exclusion at this stage were recorded by the same groups of researchers. Any disagreements regarding study inclusion were resolved through discussion among the review team.

**Fig. 1:**
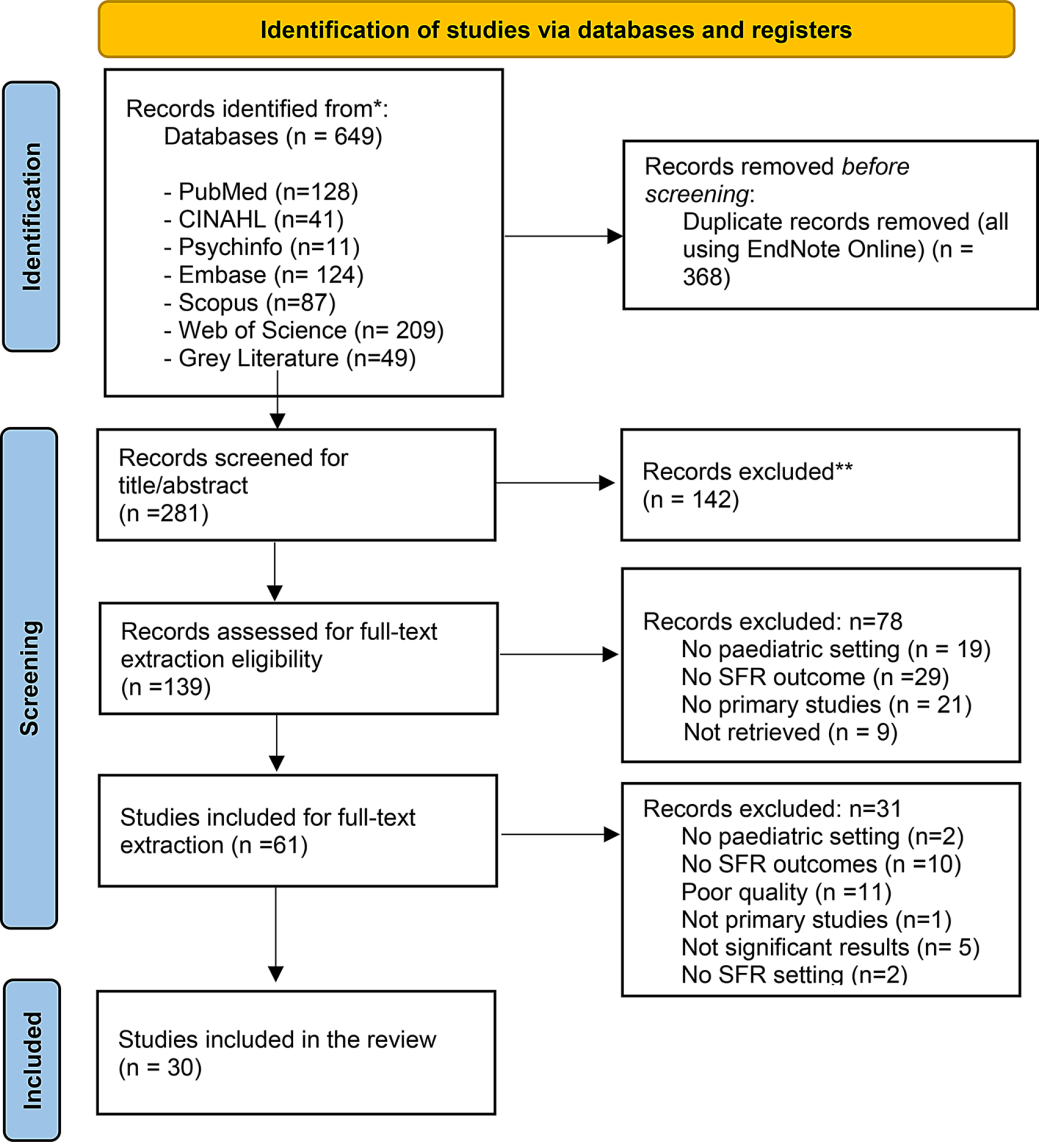
PRISMA 2020 flow diagram *Consider, if feasible to do so, reporting the number of records identified from each database or register searched (rather than the total number across all databases/registers). **If automation tools were used, indicate how many records were excluded by a human and how many were excluded by automation tools. *From*: Page MJ, McKenzie JE, Bossuyt PM, Boutron I, Hoffmann TC, Mulrow CD, et al. The PRISMA 2020 statement: an updated guideline for reporting systematic reviews. BMJ 2021;372:n71. doi: 10.1136/bmj.n71. For more information, visit: http://www.prisma-statement.org/

A detailed account of the studies excluded in the review process and reasons for exclusion is documented in PRISMA [18] diagram (Fig. 1).

### Data extraction

Three reviewers (GO, SR, ND) independently conducted the critical full-text evaluation and data extraction using a specific Data Extraction Table on Excel sheet. To test the quality of the table and the data extraction process, three authors independently extracted the data from the first three studies (in alphabetical order). After that, the authors came together to share and determine whether their approaches of the data extraction were consistent with each other’s output and whether the content of the table was in line with the research question. No reason was found to modify the data extraction tables or planned process. After a consensus of the data extraction process was reached, the data were extracted by the three independent reviewers. They then met to discuss their findings and reach a final agreement for included studies. In cases of disagreement that could not be resolved through discussion, a further reviewer was consulted for the final evaluation.

This data extraction table included the following elements: Author and Year, Country, Study Aim, Study design, Study population, Sampling, Sample size, Participants, Setting, Data Collection, Data analysis, Outcome measurements tools, Key findings, Patient outcomes, Family outcomes, Healthcare staff outcomes, Organizational outcomes, Conclusions, Limitations and Quality Appraisal.

The JBI’s Critical Appraisal Tools [19] (specific JBI’s tool for each type of study reviewed) were used to evaluate the quality of the included studies.

### Data synhtesis

The three reviewers mentioned above performed the narrative synthesis to describe the key findings and identify gaps in the literature. The narrative synthesis utilized words and text to describe study characteristics, context, quality, and results. To facilitate comparison of the extracted data, tables developed with information in the data extraction Excel table are presented in the sections below [20].

## Results

### Search results

From six different electronic databases, 649 citations were identified as being potentially relevant to the review. Then, 368 duplicates were removed by an automation tool (EndNote Online). The titles and abstracts of 281 citations were reviewed, and a total of 139 records were assessed for full text extraction eligibility. A total of 61 studies were included for full text extraction. Based on the eligibility criteria, 30 studies were critically appraised and deemed suitable for inclusion in the review. The search results and selection process are presented in the PRISMA [18] flow diagram (Fig. 1) while key findings and study characteristics are described below and in Tables 2 and 3.

**Table 2:** Characteristics of included studies

Author(s) (Year)	Country	Design/Study type	Population	Setting
Cone et al., 2010	USA	Cross-sectional survey-based study	107 NICU staff members (nurses, physicians, respiratory therapists, and other support personnel)	NICU
Doede & Trinkoff, 2020	USA	Qualitative interpretive description	15 neonatal nurses	NICU
Domanico et al., 2010	USA	Prospective study	NICU parents	NICU
Fay et al., 2023	USA	Pre-/post-occupancy evaluation with a multimethodological design using staff surveys, observations, and focus groups.	NICU Staff members (81 pre and 131 post)	NICU
Grundt et al., 2021	Norway	Longitudinal, prospective, comparative, observational study	77 Infant and 66 mothers	NICU with SFR and OB
Joshi et al., 2018	The Netherlands	Retrospective comparative	NICU patients (SFR 158; OB 170)	Two NICUs
Kainiemi et al., 2021	Finland	Pre–post study	Parents of preterm infants born before 35 weeks of gestation and admitted to the NICU	NICU
Kudchadkar et al., 2016	USA	A cross-sectional study	PICU nurses	PICU
Lester et al., 2014	USA	Longitudinal, prospective, quasi-experimental cohort study	403 NICU patients 151 OB; 252 SFR)	NICU
Lester et al., 2016	USA	Longitudinal, follow-up, quasi-experimental cohort study	216 NICU patients (93 OB; 123 SFR)	NICU
Machry et al., 2023	USA	Qualitative, ethnographic observational study	NICU families (20) and (30) healthcare staff	Two NICUs
Pickler et al., 2013	USA	Secondary analysis	87 preterm between 30–32 weeks gestation	OB and SFR NICU
Pineda et al., 2017	USA	Observational longitudinal study	58 preterm infants born at ≤ 28 weeks	NICU
Pineda et al., 2014	USA	Prospective longitudinal cohort study	136 preterm infants born < 30 weeks	NICU
Silva et al., 2024	Brazil	Comparative observational study	40 preterm neonates 20 in the Open-Bay NICU (OB NICU) 20 in the Single-Family Room NICU (SFR NICU)	NICU
Smith et al., 2009	USA	Mixed method study	NICU nurses	NICU
Solís-García et al., 2024	Spain	Observational and multi- centre study	63 neonatal units in 17 Spanish Regions: 17 (27%) were IIIA, 35 (56%) were IIIB and 11 (17%) were IIIC.	NICU
Soni et al., 2022	Qatar, Middle EAst	Descriptive study	Healthcare staff, including nurses, physicians, advanced neonatal nurse practitioners, respiratory therapists, allied health professionals	NICU
Stevens et al., 2010	USA	Comparative study	NICU staff, including nurses, neonatologists, nurse practitioners, clinical staff, non-clinical staff, pediatricians, and pediatric specialists	NICU
Tanberg et al., 2018	Norway	Case control study	NICU patients (29SFR; 31 OB)	OB and SFR NICU
Tandberg et al., 2019a	Norway	Case control study	NICU parents (60 SFR, 72 OB)	OB and SFR NICU
Tandberg et al., 2019b	Norway		NICU patients (35 SFR; 42 OB) and parents (60SFR; 72 OB)	NICU
Toivonen et al., 2017	Finland	Comparative observational study	20 NICU nurses	NICU
van der Hoeven et al., 2022	The Netherlands	Retrospective cohort study	NICU patients (1433)	NICU
van der Hoeven et al., 2023	The Netherlands	Retrospective cohort study	NICU patients (OB 1236; SFR 777)	NICU
van Veenendaal, 2020	The Netherlands	Retrospective before-after study	1046 NICU patients (468 SFR; 578 OB)	NICU
Vohr et al., 2017	USA	Retrospective cohort study	691 Preterm infants weighing ≤ 1250 g at birth (394 OB, 297 SFR)	NICU
Watson et al., 2014	Canada	Descriptive study	Healthcare NICU staff (171) and parents (173)	NICU
Wielenga et al., 2025	The Netherlands	Observational longitudinal cohort study	833 dyads of parents and infants (365 were hospitalised in the OBU phase, 140 in the transition phase, and 328 in the SFR phase)	NICU
Winner-Stoltz et al., 2018	USA	Prospective cohort study	64 NICU staff RNs full-time, part-time	NICU

**Table 3:** Outcome for included studies

Author(s) (Year)	Patient outcome	Family outcome	Staff outcome	Organizational outcome
Cone et al., 2010	x	x	x	x
Doede & Trinkoff, 2020		x	x	
Domanico et al., 2010	x	x	x	
Fay et al., 2023			x	x
Grundt et al., 2021	x	x		
Joshi et al., 2018			x	
Kainiemi et al., 2021	x	x	x	x
Kudchadkar et al., 2016	x		x	
Lester et al., 2014	x	x	x	X
Lester et al., 2016	x	x		x
Machry et al., 2023		x	x	x
Pickler et al., 2013	x			
Pineda et al., 2017	x	x		x
Pineda et al., 2014	x	x		x
Silva et al., 2024	x	x		
Smith et al., 2009		x	x	
Solis- Garcia, 2024		x		
Soni et al., 2022	x	x	x	x
Stevens et al., 2010	x	x	x	x
Tanberg et al., 2018	x	x	x	
Tandberg et al., 2019a		x		
Tandberg et al., 2019b	x			
Toivonen et al., 2017	x	x	x	x
van der Hoeven et al., 2022	x	x	x	X
van der Hoeven et al., 2023	x	x	x	x
van Veenendaal, 2020	x	x	x	x
Vohr et al., 2017	x	x	x	x
Watson et al., 2014		x	x	x
Wielenga et al., 2025	x	x		
Winner-Stoltz et al., 2018			x	x

### Key findings from included studies

This review included 30 studies conducted between 2009 and 2025 across North America, Europe, the Middle East and one in South America. Included studies employed different methodologies, with prospective cohort and observational designs being most common, enabling assessment of clinical, developmental, and experiential outcomes over time [21–23]. Retrospective cohort studies were also frequently used to analyse existing clinical data [24–26]. Several studies adopted qualitative or ethnographic approaches to explore staff and parental experiences in depth [11, 27]. Mixed-method and pre-/post-designs combined surveys, observations, and interviews to evaluate changes before and after SFR implementation [28, 29]. Lastly, cross-sectional surveys provided snapshots of perceptions and satisfaction [8, 30]. Overall, the dominant use of cohort designs was complemented by qualitative insights, offering a comprehensive understanding of SFR impacts.

Study populations comprised NICU staff, parents, and preterm infants across both single-family room (SFR) and open-bay (OB) settings.

Outcomes were classified into four main domains: patient, family, staff, and organisation. Most studies reported improvements in patient outcomes—including neurodevelopment, breastfeeding, and reduced length of stay—in SFRs compared to OBs [21, 23, 26].

Single-family rooms (SFRs) in NICUs enhance neurodevelopmental outcomes and support family-centred care [8, 9, 30], offering greater privacy, improved parental involvement, and better environmental control [31, 32, 40, 41]. SFRs are associated with earlier breastfeeding [22], fewer alarms [24], improved sleep [15], shorter hospital stays [37], and enhanced cognitive outcomes [26]. Nurses report increased job satisfaction and efficiency [21, 28], though concerns about professional isolation and teamwork persist [28, 30, 33]. While infection rates are comparable, hand hygiene adherence is higher in SFRs [34], with fewer sepsis cases and catheter use [23]. Parents experience greater emotional support and involvement [32, 35], but nurse concerns about declining parental trust remain. Although concerns about sensory deprivation and delayed maturation exist [36], overall, SFRs contribute to better developmental care, workflow efficiency, and long-term cost-effectiveness [26, 31].

### Patient outcomes

The SFR environment generally supports neonatal neurodevelopment by providing a quieter [40, 41], more controlled setting that reduces noise exposure [33, 36]. Infants in SFRs show improved sleep regulation and circadian rhythms [15], along with enhanced parent-infant closeness, privacy, skin-to-skin contact, communication with medical staff, and easier parental access [8, 29, 42] promoting parent-infant bonding, and contributing to more supportive developmental care [42].

Clinical outcomes are also improved in SFR settings. Infants experience shorter lengths of stay (LOS) and reduced mortality rates [23, 33], better growth including higher discharge weight and faster weight gain, earlier attainment of full enteral feeding at lower gestational ages, improved biobehavioral regulation and reduced stress levels [40]. They undergo fewer medical procedures, have lower sepsis incidence, and demonstrate improved neurobehavioral outcomes, such as reduced pain, stress, and hypertonicity [21]. Cognitive and language development at 18 months is enhanced, with a decreased risk of autism spectrum disorder symptoms [37]. Furthermore, exclusive breastfeeding rates at discharge and up to four months are higher in SFR infants [22].

However, some concerns remain. Infants in SFRs have shown reduced hemispheric asymmetry and delayed cerebral maturation on EEG, with lower language and motor scores at two years, possibly due to sensory deprivation in a less stimulating environment [36]. Infection control also requires attention, as cases of infection among twins suggest simultaneous acquisition rather than cross-transmission [34].

Parental involvement is significantly increased in SFRs, with mothers and fathers engaging in an additional eight and 37 h of skin-to-skin contact, respectively, over the hospital stay [32]. These factors contribute to enhanced patient safety and improved long-term neurodevelopmental outcomes [26, 31]. Overall, these findings support implementing SFRs in NICUs to optimize developmental, clinical, and psychosocial outcomes.

### Family outcomes

SFRs in NICUs provide enhanced privacy, allowing families increased time with their infants and greater involvement in caregiving activities [11, 23, 29, 30, 33]. Parents report improved comfort and a heightened sense of environmental control within SFRs, which facilitates active participation in infant care [33, 42] contributing to reductions in parental stress and improved emotional outcomes [40]. However, the increased privacy inherent to SFRs may limit interparental communication and social interaction, potentially contributing to parental isolation [8, 33].

Quantitative data indicate significantly higher parental presence in SFRs, with mothers and fathers spending an additional 13 and 4 h per day at the bedside, respectively [12, 27, 32, 36]. This increased presence correlates with earlier initiation of milk expression and breastfeeding, supported by enhanced lactation resources and breastfeeding support programs [22, 26]. Maternal involvement in kangaroo care and feeding practices is also increased, accompanied by reductions in maternal stress and enhanced opportunities for skin-to-skin contact [21, 22, 36, 37, 40]. Fathers similarly benefit from increased skin-to-skin contact during the infant’s hospitalization [32].

Parental emotional well-being appears improved in SFR environments, with mothers reporting lower stress levels and decreased prevalence of depressive symptoms compared to those in open-bay units [9, 21, 35]. Environmental stressors such as noise and light exposure are also reduced in SFRs, further supporting parental mental health [38]. Enhanced parental engagement [42] fosters greater confidence in caregiving abilities and contributes to improved adjustment following discharge [9, 12]. Furthermore, SFRs facilitate improved communication between parents and healthcare providers, resulting in increased parental trust and involvement in clinical decision-making, although some nursing staff express concerns regarding diminished professional trust due to greater family autonomy [32].

### Healthcare staff outcomes

SFRs contribute to a more structured and quieter work environment, which staff generally perceive as beneficial [30]. However, reduced peer interaction in SFRs often leads to feelings of isolation among staff, negatively affecting teamwork and collegial support [8, 28, 30]. Despite this, nurses acknowledge that SFRs enhance family-centred care (FCC) by enabling greater focus on individualized infant care and parental support [27, 30].

The emotional burden on nurses increases in SFRs, particularly when compensating for limited parental involvement, often due to social challenges, which can heighten stress levels [11, 33]. Nurses transitioning to SFRs initially report increased stress and concerns about patient safety but tend to experience improved job satisfaction over time [15, 21, 28, 33].

Overall, staff report higher job satisfaction in SFRs, with lower stress and reduced alarm fatigue contributing to better well-being [15, 24]. Nurses spend more time engaging with parents and providing lactation support, reinforcing their role in FCC. For example, in one study is reported that nurse-infant interaction increases from 119 to 166 min per shift and the number of interactions from 22 to 26 per shift in SFRs compared to open-bay (OB) units. Similarly, nurse-family interaction time rises from 35 to 117 min per shift, with interactions increasing from 8 to 13 per shift [12].

Challenges remain, including increased physical demands [27], communication difficulties with staff, and fewer opportunities for peer support [8, 33]. Conversely, some studies report improved physical work environments in SFRs, characterized by lower noise levels, greater privacy, fewer interruptions [31], and enhanced concentration [9]. Staff also express greater satisfaction with both physical and emotional aspects of the workspace [2].

SFRs positively influence infection control, with nurses demonstrating higher hand hygiene compliance [25]. Nonetheless, concerns about reduced teamwork and potential declines in trust-building between nurses and families persist [32].

### Organizational outcomes

From an institutional perspective, SFRs offer multiple advantages, including enhanced infection control and increased compliance with hygiene protocols, which contribute to improved patient safety [25, 30]; Although concerns exist regarding multidrug-resistant organism (MDRO) colonization, evidence suggests these infections arise from external introduction rather than intra-unit transmission [34].

SFR implementation is associated with greater workflow efficiency, improved access to supplies, better workspace utilization [28], and more streamlined communication [25]. However, design elements can act as both facilitators and barriers to SFR effectiveness [27]. While transitioning to SFRs necessitates additional staff training, these environments ultimately enhance developmental care and support stronger adherence to FCC principles [12, 21, 31].

A key benefit of SFRs is the potential for reduced hospital costs. Shorter lengths of stay observed in infants cared for in SFRs lead to fewer medical interventions and lower healthcare expenses [23, 37]. Furthermore, improved developmental outcomes associated with SFRs may yield long-term cost savings by decreasing the need for future medical and educational services [26].

Despite these improvements in care quality and efficiency, SFRs have not been shown to significantly affect staff sick leave, turnover, or injury rates [9].

## Discussion

This mixed-methods systematic review contributes to the growing body of evidence supporting the benefits of SFR designs in NICUs, while highlighting the paucity of research in PICU settings. Given the distinct clinical profiles and care demands of NICU versus PICU patients, this gap underscores an urgent need for studies assessing the impact of SFRs in older paediatric populations, staff workflows, and family engagement within high-acuity environments.

In line with previous research [2–4], our findings reinforce that SFRs promote family-centred care (FCC), enhance infant neurodevelopment, improve maternal and parental well-being, and optimize healthcare delivery. Enhanced parent-infant bonding, increased skin-to-skin contact, and earlier breastfeeding initiation were recurrently observed, affirming the critical role of parental presence in neonatal development [1, 6]. These improvements correlate with better clinical outcomes, including improved sleep regulation, reduced pain and stress responses, and shorter hospital stays [21, 26, 33]. From a developmental care standpoint, environmental regulation—through controlled light, sound, and minimized disruptions—emerges as central in supporting preterm infants’ physiological and neurological stability [36]. However, the review also echoes concerns from earlier work [4, 13] about the potential for sensory deprivation in highly isolated SFR environments due to reduced auditory and social stimulation. This has been associated with delayed cerebral maturation and lower language and motor scores at two years of age. These findings emphasize the need to carefully balance sensory exposure in SFRs—ensuring that the environment remains developmentally appropriate while minimizing harmful stimuli. Incorporating structured developmental stimulation programs may help address this balance and warrants further research.

From the staff perspective, SFRs are perceived to improve the physical work environment, reduce alarm fatigue, and facilitate enhanced family interactions, aligning with reported increases in job satisfaction and quality of care [2, 9]. Nevertheless, challenges remain, including increased emotional labour, feelings of isolation, and diminished team cohesion, especially when parental involvement is limited [11, 30]. These findings suggest that while SFRs support FCC principles, they may inadvertently hinder staff collaboration unless supplemented by organizational interventions such as team-building initiatives, peer support mechanisms, and targeted training programs.

Organizationally, evidence indicates improved workflow efficiency, better infection control adherence, and reduced costs linked to shorter lengths of stay in SFR units, corroborating prior claims of operational benefits associated with SFR implementation [5, 7]. While concerns about multidrug-resistant organism (MDRO) colonization persist, current data suggest colonization primarily arises from community rather than in-unit transmission [34]. Effective infection control practices and staff adherence to hygiene protocols remain critical components of successful SFR adoption.

Equity considerations also emerge as essential. Socioeconomic factors significantly influence parental presence and engagement, affecting both infant developmental outcomes and parental well-being [26]. Future research should explore how institutional policies—such as provision of housing, transportation assistance, and parental leave—can mitigate disparities and ensure equitable access to SFR benefits. Broader inclusion of diverse caregiver populations, including those facing cultural, linguistic, or systemic barriers, is also critical. Moreover, it is plausible that infants whose parents are unable to be present in SFRs due to personal, social, or structural constraints may experience fewer benefits from family-centred care. To mitigate the risk of widening disparities, healthcare institutions might consider implementing strategies such as enhanced nursing presence or structured care programmes to ensure consistent developmental and emotional support. Further research is needed to explore how best to safeguard equitable care experiences for this potentially vulnerable subgroup.

Advancements in technology and artificial intelligence (AI) hold promise for mitigating some of the structural and interpersonal challenges associated with SFR designs. AI-driven monitoring systems could reduce alarm fatigue by providing more precise and context-aware alerts, allowing nurses to focus their attention more efficiently [24]. Telehealth platforms and digital communication tools may facilitate improved collaboration and information sharing among dispersed staff members, helping to overcome the isolation and reduced peer interaction inherent in SFR layouts [28]. Additionally, AI-enabled predictive analytics could support individualized patient care planning, enhancing developmental care and parental involvement by providing real-time decision support tailored to each infant’s needs. These technologies could also empower families through remote access to care teams and educational resources, further strengthening FCC. However, successful integration of such innovations requires thoughtful implementation strategies, including training and workflow redesign, to ensure that technology complements rather than complicates the caregiving environment.

While the SFR model represents a significant advance in delivering developmentally sensitive, family-centred care, its optimal implementation requires coordinated structural, emotional, and organizational support. Future research, particularly in PICU settings, alongside interventions targeting staff collaboration and equity, is essential to maximize the benefits of SFR designs across paediatric intensive care environments.

### Limitation

A notable limitation of this review is the heterogeneity across included studies, encompassing variations in design, populations, outcomes, and reporting styles. This diversity precluded meta-analytic synthesis and limits causal inference. Additionally, the language restriction to English, Italian, and Spanish may have omitted pertinent evidence from other linguistic contexts. Furthermore, the predominance of observational study designs underscores the need for high-quality randomized controlled trials to strengthen the evidence base.

## Conclusions

The shift from open-bay to SFRs NICUs offers clear benefits, notably increased parental presence and engagement linked to improved infant neurodevelopment and reduced parental stress [21, 26, 29, 32, 35, 37]. However, these gains do not always result in significant clinical improvements [25]. While SFRs support earlier developmental milestones [21, 22], reduced auditory stimulation raises concerns about sensory exposure [13, 36]. Infection rates show little change after transitioning to SFRs [34].

Staff report better work environments and communication but face increased emotional demands and mixed effects on workflow efficiency [8, 11, 28]. Physical design influences staff responsiveness and alarm management, though improvements in care quality are not uniform [24, 39].

Overall, SFRs enhance family-centred care but present complex challenges. Future research should focus on refining NICU design and care models to balance infant development, parental involvement, staff well-being, and operational efficiency. Additionally, exploring technological innovations and targeted support strategies may help overcome current limitations and maximize the benefits of SFR environments.

These findings are relevant for healthcare planners and clinical leaders aiming to enhance family-centred care, staff well-being, and developmental outcomes in intensive care contexts.

## Data Availability

All data generated or analyzed during this study are included in this published article and its supplementary information files. As this is a systematic review, the data consist of previously published studies that were identified through structured database searches and are publicly available. Detailed search strategies, inclusion/exclusion criteria, and the list of included studies are provided in this article. No new raw data were generated for this review.

## Abbreviations

FCC: Family-Centred Care
NICU: Neonatal Intensive Care Unit
OB: Open Bay
PICU: Pediatric Intensive Care Unit
SFR: Single-Family Room

## References

[1] European Foundation for the Care of Newborn Infants. The NICU of the future: design meets function [Internet]. 2019. Available from: https://www.efcni.org/news/the-nicu-of-the-future-design-meets-function

[2] Stevens DC, Helseth CC, Khan MA, Munson DP, Smith TJ. Neonatal intensive care nursery staff perceive enhanced workplace quality with the single-family room design. J Perinatol. 2010;30(5):352–8. 10.1038/jp.2009.137. 19798047 10.1038/jp.2009.137

[3] Smith TJ, Schoenbeck K, Clayton S. Staff perceptions of work quality of a neonatal intensive care unit before and after transition from an open Bay to a private room design. Work. 2009;33(2):211–27. 10.3233/WOR-2009-0868. 19713631 10.3233/WOR-2009-0868

[4] Meredith JL, Jnah A, Newberry D. The NICU environment: infusing single-family room benefits into the open-bay setting. Neonatal Netw. 2017;36(2):69–76. Available from: 10.1891/0730-0832.36.2.69 10.1891/0730-0832.36.2.69 28320493

[5] White RD, Consensus Committee on Recommended Design Standards for Advanced Neonatal Care. Recommended standards for newborn ICU design, 9th edition. J Perinatol. 2020;40(Suppl 1):2–4. Available from: 10.1038/s41372-020-0766-2 10.1038/s41372-020-0766-2 32859957

[6] Alsadaan N, Ramadan OME, Alqahtani M, Shaban M, Elsharkawy NB, Abdelaziz EM, et al. Impacts of integrating family-centered care and developmental care principles on neonatal neurodevelopmental outcomes among high-risk neonates. Children (Basel). 2023;10(11):1751. Available from: 10.3390/children10111751 10.3390/children10111751 PMC10670637 38002842

[7] Ortenstrand A, Westrup B, Broström EB, Sarman I, Akerström S, Brune T, et al. The Stockholm neonatal family-centered care study: effects on length of stay and infant morbidity. Pediatrics. 2010;125(2):e278–85. Available from: 10.1542/peds.2009-1511 10.1542/peds.2009-1511 20100748

[8] Soni R, Fairhurst N, El Anbari M, Leslie A, Tscherning Wel-Wel C. Staff perceptions and challenges of the single-family room design: experience of a greenfield level 4 neonatal intensive care unit in the middle East. Acta Paediatr. 2022;111(12):2291–8. 10.1111/apa.16527. 36017578 10.1111/apa.16527

[9] Watson J, DeLand M, Gibbins S, MacMillan York E, Robson K. Improvements in staff quality of work life and family satisfaction following the move to single-family room NICU design. Adv Neonatal Care. 2014;14(2):129–36. 10.1097/ANC.0000000000000046. 24675633 10.1097/ANC.0000000000000046

[10] Kaur H, Rohlik GM, Nemergut ME, Tripathi S. Comparison of staff and family perceptions of causes of noise pollution in the pediatric intensive care unit and suggested intervention strategies. Noise Health. 2016;18(81):78–84. 10.4103/1463-1741.178480. 26960784 10.4103/1463-1741.178480 PMC4918686

[11] Doede M, Trinkoff AM. Emotional work of neonatal nurses in a single-family room NICU. J Obstet Gynecol Neonatal Nurs. 2020;49(3):283–92. Available from: 10.1016/j.jogn.2020.03.001 10.1016/j.jogn.2020.03.001 32298637

[12] Toivonen M, Lehtonen L, Löyttyniemi E, Axelin A. Effects of single-family rooms on nurse-parent and nurse-infant interaction in neonatal intensive care unit. Early Hum Dev. 2017;106–107:59–62. 10.1016/j.earlhumdev.2017.01.012. 28199954 10.1016/j.earlhumdev.2017.01.012

[13] Pineda RG, Neil J, Dierker D, Smyser CD, Wallendorf M, Kidokoro H, et al. Alterations in brain structure and neurodevelopmental outcome in preterm infants hospitalized in different neonatal intensive care unit environments. J Pediatr. 2014;164(1):52–e602. 10.1016/j.jpeds.2013.08.047. 24139564 10.1016/j.jpeds.2013.08.047 PMC3872171

[14] Rand K, Lahav A. Impact of the NICU environment on Language deprivation in preterm infants. Acta Paediatr. 2014;103(3):243–8. 10.1111/apa.12481. 24164604 10.1111/apa.12481

[15] Kudchadkar SR, Beers MC, Ascenzi JA, Jastaniah E, Punjabi NM. Nurses’ perceptions of pediatric intensive care unit environment and work experience after transition to single-patient rooms. Am J Crit Care. 2016;25(5):e98–107. 10.4037/ajcc2016463. 27587429 10.4037/ajcc2016463

[16] Weigel DJ. The concept of family: an analysis of laypeople’s views of family. J Fam Issues. 2008;29(11):1426–48. Available from: 10.1177/0192513X08318488

[17] Ottonello G, Rossi S, Dasso N, Da Rin Della Mora R, Calza S, Caracciolo Minniti G, Artuso I, Serveli S, Esibiti F, Rebuffi C, Parodi S, Scelsi S. The effects of the introduction of the single-family room in neonatal intensive care and paediatric intensive care on the outcomes of paediatric patients, families, staff, and organizations: a mixed method systematic review protocol. Scenario. 2025;42(2):616. (Article in Production). 10.1186/s12913-025-13595-8 PMC12551196 41136975

[18] Page MJ, McKenzie JE, Bossuyt PM, Boutron I, Hoffmann TC, Mulrow CD, et al. The PRISMA 2020 statement: an updated guideline for reporting systematic reviews. Syst Reviews. 2021;10(1):1–11. 10.1186/s13643-021-01626-4 PMC8008539 33781348

[19] Johanna Briggs Institute. JBI critical appraisal tools [Internet]. Available from: https://jbi.global/critical-appraisal-tools

[20] Hannes K, Lockwood C. Synthesizing qualitative research: choosing the right approach. Hoboken (NJ): John Wiley & Sons; 2012. Available from: 10.1002/9781119959847

[21] Lester BM, Hawes K, Abar B, Sullivan M, Miller R, Bigsby R, et al. Single-family room care and neurobehavioral and medical outcomes in preterm infants. Pediatrics. 2014;134(4):754–60. 10.1542/peds.2013-4252. 25246623 10.1542/peds.2013-4252

[22] Grundt H, Tandberg BS, Flacking R, Drageset J, Moen A. Associations between single-family room care and breastfeeding rates in preterm infants. J Hum Lact. 2021;37(3):593–602. Available from: 10.1177/0890334420962709 10.1177/0890334420962709 PMC8414820 33035125

[23] van Veenendaal NR, van der Schoor SRD, Heideman WH, Rijnhart JJM, Heymans MW, Twisk JWR, et al. Family integrated care in single family rooms for preterm infants and late-onset sepsis: a retrospective study and mediation analysis. Pediatr Res. 2020;88(4):593–600. 10.1038/s41390-020-0875-9. 32241017 10.1038/s41390-020-0875-9

[24] Joshi R, van Straaten H, van Mortel H, Long X, Andriessen P, van Pul C. Does the architectural layout of a NICU affect alarm pressure? A comparative clinical audit of a single-family room and an open Bay area NICU using a retrospective study design. BMJ Open. 2018;8(6):e022813. 10.1136/bmjopen-2018-022813. 29961040 10.1136/bmjopen-2018-022813 PMC6045752

[25] van der Hoeven A, Bekker V, Jansen SJ, Saccoccia B, Berkhout RJM, Lopriore E, et al. Impact of transition from open Bay to single room design neonatal intensive care unit on multidrug-resistant organism colonization rates. J Hosp Infect. 2022;120:90–7. 10.1016/j.jhin.2021.12.006. 34902498 10.1016/j.jhin.2021.12.006

[26] Vohr B, McGowan E, McKinley L, Tucker R, Keszler L, Alksninis B. Differential effects of the single-family room neonatal intensive care unit on 18- to 24-month Bayley scores of preterm infants. J Pediatr. 2017;185:42–e481. 10.1016/j.jpeds.2017.01.056. 28238479 10.1016/j.jpeds.2017.01.056

[27] Machry H, Joseph A, White R, Allison D. Designing for family engagement in neonatal icus: how is the interior design of single-family rooms supporting family behaviors. Passive active? HERD. 2023;16(3):238–60. 10.1177/19375867231168651. 37157783 10.1177/19375867231168651

[28] Fay L, Real K, Haynes S, Daneshvar Z. Examining efficiency in open-bay and single-family room NICU designs. Adv Neonatal Care. 2023;23(4):355–64. Available from: 10.1097/ANC.0000000000001058 10.1097/ANC.0000000000001058 36719284

[29] Kainiemi E, Hongisto P, Lehtonen L, Pape B, Axelin A. Effects of single family room architecture on parent-infant closeness and family centered care in neonatal environments: a single-center pre-post study. J Perinatol. 2021;41(9):2244–51. 10.1038/s41372-021-01137-z. 34230604 10.1038/s41372-021-01137-z PMC8440171

[30] Cone SK, Short S, Gutcher G, Zhong Y. From baby barn to the single family room designed NICU: a report of staff perceptions one year post occupancy. Newborn Infant Nurs Rev. 2010;10(2):97–103. Available from: 10.1053/j.nainr.2010.03.002

[31] Winner-Stoltz R, Lengerich A, Hench AJ, O’Malley J, Kjelland K, Teal M. Staff nurse perceptions of open-pod and single family room NICU designs on work environment and patient care. Adv Neonatal Care. 2018;18(3):189–98. 10.1097/ANC.0000000000000493. 29794838 10.1097/ANC.0000000000000493

[32] Tandberg BS, Frøslie KF, Flacking R, Grundt H, Lehtonen L, Moen A. Parent-infant closeness, parents’ participation, and nursing support in single-family room and open Bay nicus. J Perinat Neonatal Nurs. 2018;32(4):E22–32. 10.1097/JPN.0000000000000359. 30358674 10.1097/JPN.0000000000000359

[33] Domanico R, Davis DK, Coleman F, Davis BO Jr. Documenting the NICU design dilemma: parent and staff perceptions of open ward versus single family room units. J Perinatol. 2010;30(5):343–51. Available from: 10.1038/jp.2009.195 10.1038/jp.2009.195 PMC2864417 20072132

[34] van der Hoeven A, Jansen SJ, Kraakman M, Bekker V, Veldkamp KE, Boers SA, et al. Influence of transition from open Bay units to single room units in a neonatal intensive care unit on hospital transmission of multi-drug-resistant enterobacterales. J Hosp Infect. 2023;141:3–8. 10.1016/j.jhin.2023.07.026. 37611696 10.1016/j.jhin.2023.07.026

[35] Flacking R, Markestad T, Grundt H, Moen A. Parent psychological wellbeing in a single-family room versus an open Bay neonatal intensive care unit. PLoS ONE. 2019;14(11):e0224488. 10.1371/journal.pone.0224488. 31689307 10.1371/journal.pone.0224488 PMC6830777

[36] Pineda R, Durant P, Mathur A, Inder T, Wallendorf M, Schlaggar BL. Auditory exposure in the neonatal intensive care unit: room type and other predictors. J Pediatr. 2017;183:56–e663. 10.1016/j.jpeds.2016.12.072. 28189301 10.1016/j.jpeds.2016.12.072 PMC5378448

[37] Lester BM, Salisbury AL, Hawes K, Dansereau LM, Bigsby R, Laptook A, et al. 18-month follow-up of infants cared for in a single-family room neonatal intensive care unit. J Pediatr. 2016;177:84–9. 10.1016/j.jpeds.2016.06.069. 27470693 10.1016/j.jpeds.2016.06.069

[38] Tandberg BS, Frøslie KF, Markestad T, Flacking R, Grundt H, Moen A. Single-family room design in the neonatal intensive care unit did not improve growth. Acta Paediatr. 2019;108(6):1028–35. 10.1111/apa.14746. 30729563 10.1111/apa.14746

[39] Pickler RH, McGrath JM, Reyna BA, Tubbs-Cooley HL, Best AM, Lewis M, et al. Effects of the neonatal intensive care unit environment on preterm infant oral feeding. Res Rep Neonatol. 2013;3:15–20. 10.2147/RRN.S41280. 10.2147/RRN.S41280 PMC4278377 25552910

[40] Silva NF, Linhares MBM, Gaspardo CM. Stress and self-regulation behaviors in preterm neonates hospitalized at open-bay and single-family room neonatal intensive care unit. Infant Behav Dev. 2024;76:101951. 10.1016/j.infbeh.2024.101951. 38663037 10.1016/j.infbeh.2024.101951

[41] Solís-García G, Cambra-Rufino L, Piris Borregas S, Carrasco Pérez A, López Maestro M, De la Cruz Bértolo J, et al. Architectural design, facilities and family participation in neonatal units in spain: A multicentre study. Acta Paediatr. 2024;113(4):716–21. 10.1111/apa.17085. 38186235 10.1111/apa.17085

[42] Wielenga JM, Pascual A, Ruhe K, Aarnoudse C, van Kaam AH. Effect of shifting from open Bay to Single-Family rooms on closeness in a NICU. Acta Paediatr. 2025. 10.1111/apa.70108. Epub ahead of print. 40264380 10.1111/apa.70108 PMC12336955

